# Implications of hematopoietic stem cells heterogeneity for gene therapies

**DOI:** 10.1038/s41434-021-00229-x

**Published:** 2021-02-15

**Authors:** Jeremy Epah, Richard Schäfer

**Affiliations:** Institute for Transfusion Medicine and Immunohaematology, German Red Cross Blood Donor Service Baden-Württemberg-Hessen gGmbH, Goethe University Hospital, Frankfurt am Main, Germany

**Keywords:** Hematopoietic stem cells, Gene therapy, Heterogeneity, Subpopulation, Lineage, Cell biology, Diseases

## Abstract

Hematopoietic stem cell transplantation (HSCT) is the therapeutic concept to cure the blood/immune system of patients suffering from malignancies, immunodeficiencies, red blood cell disorders, and inherited bone marrow failure syndromes. Yet, allogeneic HSCT bear considerable risks for the patient such as non-engraftment, or graft-versus host disease. Transplanting gene modified autologous HSCs is a promising approach not only for inherited blood/immune cell diseases, but also for the acquired immunodeficiency syndrome. However, there is emerging evidence for substantial heterogeneity of HSCs in situ as well as ex vivo that is also observed after HSCT. Thus, HSC gene modification concepts are suggested to consider that different blood disorders affect specific hematopoietic cell types. We will discuss the relevance of HSC heterogeneity for the development and manufacture of gene therapies and in exemplary diseases with a specific emphasis on the key target HSC types myeloid-biased, lymphoid-biased, and balanced HSCs.

## Introduction

It was 30 years ago when the first gene therapy had started transfusing autologous T cells that were retrovirally transduced to produce adenosine deaminase (ADA) to two girls aged 4 and 9 years, respectively, with severe combined immunodeficiency due to ADA shortage (ADA-SCID) [1, 2]. Since then, gene therapy strategies have been developed not only for primary immunodeficiencies, but also for red blood cell (RBC) disorders and inherited bone marrow (BM) failure syndromes [3–6], thus inspiring the gene therapy field and giving hope to patients suffering from genetic blood cell disorders who were otherwise depending on a matched BM transplant, or on a matched apheresis product containing allogeneic hematopoietic stem cells (HSCs) being mobilized into the peripheral blood.

The concept to substitute the organism specifically with the corrected cell type being involved in the disease, e.g. T cells, appeared intriguing particularly when these cells could be easily harvested from the patient and genetically manipulated such as by vector-driven introduction of the normal *ADA* gene [7]. Yet, analysis of the clinical courses of both pioneering patients after a 12 years observation period showed a mixed picture. On the one hand, no adverse events were recorded suggesting the safety of the concept; on the other hand, both patients were still depending on external ADA substitution, and the persistence of the retroviral gene in the patients’ lymphocytes after repetitive applications of transduced T cells was highly variable between 20% in one patient and below 1% in the other patient who developed a humoral response against the retroviral envelope and lipoprotein components of the fetal calf serum that was used for the ex vivo culture of the autologous cells [1]. HSC transplantations (HSCT) have been performed for decades to reconstitute the blood/immune system after eradication of the diseased blood/immune cells [8]. Thus, as an alternative to terminally differentiated hematopoietic cells with limited circulation time and short life span as well as highly specialized functions, HSCs, being able to give rise to all blood cells, were utilized for gene therapy vehicles.

The terminologies defining hematopoietic “stem cell” or “progenitor cell” activities have been evolved over the past decades, and they are still being inconsistently used today. In the following the term “HSCs” is applied to cells that can give rise to all blood cell lineages thus, by self-renewal and differentiation, maintaining complete hematopoiesis [9]. Of note, as knowledge on HSC biology has evolved over time, not all studies reported the details if they have applied the above mentioned rigorous definition for HSC. This pertains specifically to the functional assessment of the differentiation capacity into all blood cell types after transplantation. For example, some studies address myeloid and lymphoid progenitors and some of their progeny (e.g. granulocytes and B lymphocytes), but do not explicitly report erythroid or megakaryocytic cells. Nevertheless, we deemed some of these studies as relevant for the understanding of possible implications of HSC heterogeneity particularly for gene therapies, and highlighted non-reported blood cell types.

In contrast, hematopoietic progenitor cells (HPCs) states define a network of potential differentiation trajectories preceding ultimate lineage commitment [10], but without the ability of self-renewal and to maintain complete hematopoiesis over lifetime. When the implications refer to both HSCs and HPCs, the term “HSPCs” is used.

Also, to set the scientific reports into context, it is worth mentioning that some insights have been gained by nonhuman research, i.e. mainly investigating mouse models, that could not be confirmed yet for the human system. This is complicated by the fact that a reliable system for experimentally validating and, thus, defining human HSCs (self-renewal, long term repopulation, and generation of all blood lineages) is not available today. Moreover, the surface marker sets and definitions being used for HSCs and their immediate progeny are different for the mouse and human systems [11–14].

**Fig. 1:**
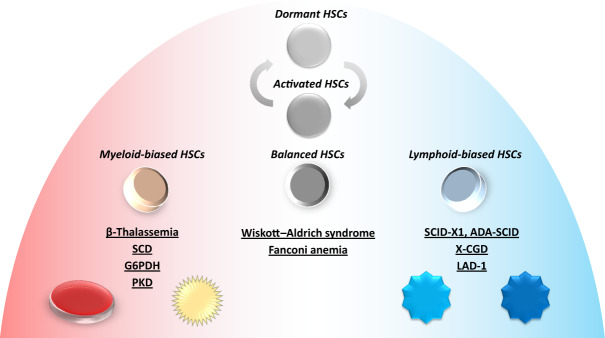
HSC subsets and gene therapies in clinical trials. SCD sickle cell disease; G6PDD glucose-6-phosphate dehydrogenase deficiency; PKD pyruvate kinase deficiency; SCID severe combined immunodeficiency X-CGD X-linked chronic granulomatous disease; LAD-1 leukocyte adhesion deficiency-1. Arrows indicate reversible phenotype shift from dormant HSCs to activated HSCs.

In the first decades BM was mainly used for HSCT yet, since the 1980s blood separation technologies improved leading to today’s modern apheresis procedures that enable the highly sufficient collection of HSCs from the peripheral blood, generally referred to as peripheral blood stem cells (PBSCs) [15], and to date both BM and PBSCs are relevant HSC sources for gene therapies [16, 17]. Currently, a variety of diseases has been targeted by HSC-based gene therapies (Fig. 1).

It seems obvious to apply allogeneic HSCs from healthy donors as such transplants would not carry the disease and, thus, would not need genetic modification to repair the patient’s blood/immune system for good. Indeed, for primary immune deficiencies, hemoglobinopathies, storage, and metabolic disorders, as well as for congenital cytopenias and stem cell defects allogeneic HSCT has been performed [18]. However, despite being established as standard treatment for hematologic malignancies such as acute leukemia or myelodysplastic syndrome, allogeneic HSCT, particularly from haplo-identical relatives or unrelated donors, bear considerable risks for the patient such as graft-versus-host disease, non-engraftment, and, not least, the risk of not finding an HLA matching donor who is willing or eligible to donate [18–20]. To mitigate the risks immunoablative strategies including anti-thymocyte globulin or Alemtuzumab have been developed yet, they come with side effects bearing additional risks for the patient [18, 21, 22]. These immunological obstacles do not apply for the patients’ own HSCs, and the conditioning regime applied prior to transplantation can be milder with less detrimental effects on the patient’s BM niche potentially making the engraftment easier [18]. Thus, genetically modified autologous HSCs appear to date as the most promising candidates for gene therapies to treat genetic blood/immune cell disorders [18]. Besides optimizing HSC collection technologies and transplantation protocols, as well as developing novel HSC-based gene therapy concepts, research has revealed more recently substantial heterogeneity amongst HSPCs [23], and shed light on the impact of pathologies on the hematopoietic niche in situ [24, 25]. Within the multipotent progenitors (MPPs) compartment lineage-biased subsets can be detected [14]. The heterogeneity on the stem cell level is specifically illustrated by the presence of balanced, myeloid- or lymphoid-biased HSCs (Fig. 1), as defined by the ratio of lymphoid to their myeloid cell progenies [26]. Of note, these findings have been reported so far only for the mouse (no erythroid or megakaryocytic cells explicitly reported). As an influencing factor human hematopoietic aging was not only shown to increase the overall frequency of (Lin− CD34+ CD38− CD90+ CD45RA−) HSCs, but also, impacting gene expression, to increase myeloid-biased HSCs in elderly individuals (no T-cells, erythroid, or megakaryocytic cells reported) [27]. In the following, we will provide an overview on the current gene therapy concepts utilizing HSCs and discuss possible implications of their heterogeneity, with an emphasis on their lineage biases, for HSC-based gene therapies.

### Heterogeneity of hematopoietic stem and progenitor cells

In the 1950s mouse studies showed that intravenous application of BM cells to lethally irradiated recipient animals restored their eradicated blood system [28]. Following these promising preclinical studies, E. Donnall Thomas performed the first successful transplantations of human BM from identical twin donors in 1959 providing evidence that systemically applied BM cells can lead to the “return of marrow function” in patients after lethal irradiation [29]. Besides the groundbreaking impact on the treatment options for these patients that has led to today’s sophisticated HSCT concepts, research has also provided insights into the blood system’s homeostasis. Hallmark studies identified clonal (colony forming) hematopoietic cells in the BM and in the mouse spleen with the potential to generate various hematopoietic cell types such as lymphocytes, granulocytes, RBCs, and megakaryocytes [30–32]. These observations prompted the concept of HSCs that can not only differentiate into all blood cell types, but have also the ability to keep their stemness (self-renewal) [32]. During the quest to prove the existence and to characterize HSCs, advanced mouse models for competitive transplantations and lineage (Lin)-specific antibodies that identify already committed blood cells were developed [32]. Further analyzing the Lin-negative BM cell fraction placed the long-term self-renewing HSCs (LT-HSCs) at the top of a hierarchical model of hematopoiesis as developed by Irv Weissman and others [33]. The highest long-term reconstitution potential can be assigned to so-called dormant HSCs that can be found at very low frequency in the murine and human BM [34, 35]. Yet, there is heterogeneity even within the dormant HSC pool, as illustrated by a recent study reporting that human LT-HSCs do not produce the cyclin-dependent kinase (CDK) 6, separating them from another HSC subset, that is also quiescent, but produces CDK6 and can easier enter the cell cycle [35].

In contrast to the MPPs as their progeny, the LT-HSCs can achieve the sustainable reconstitution of all blood cell types upon transplantation [32]. With increasing lineage commitment the common myeloid progenitors and common lymphoid progenitors produce, via more lineage-restricted progenitors, the terminally differentiated blood cells [32]. The identification of “the” HSC phenotype would not only allow the enrichment of HSCs for experimental and clinical applications, but also introduce a dose measure for HSCT. In the early 1980s the marker CD34 was identified on immature normal human BM cells as well as on leukemic human cells [36]. Indeed, proving the clinical applicability of this marker, data indicated that at least 2 × 10^6^ CD34+ cells per kg recipient’s body weight are needed for the engraftment of the transplant, while higher doses can speed up the process particularly for autologous HSCT [16, 37]. However, studies showed that CD34 expression on the cell surface is shared by numerous hematopoietic cell types varying from HSCs to multi/oligopotent progenitor cells and lineage-restricted progenitor cells, and can be detected even on non-hematopoietic cells [32, 38–40]. Within the human CD34-expressing cell family, a very small cell population was shown to repopulate the BM of non-obese diabetic severe combined immunodeficient mice, thus being depicted as SCID-repopulating cells (SRCs) [41]. Such SRCs are detectable at a low frequency of approximately one of six hundred cells with the CD34+ CD38− phenotype in the BM or in PBSCs [38, 41]. In these studies the repopulation capacity of SRCs was shown for granulocytes, monocytes, and B lymphocytes, but not for erythroid cells or platelets, traits that characterize, together with self-renewal potential, LT-HSCs. As an additional (LT-)HSC marker CD133 was introduced, and for both CD34 and CD133 clinical large-scale isolation technologies are available, but CD133+ selected cells featured superior ex vivo proliferation potential compared to CD34+ selected cells [38, 42], and CD133 was successfully used as entry receptor for lentiviral gene transfer into LT-HSCs [38]. Their sustainable repopulation potential suggests LT-HSCs as a prime target cell type for gene therapies [43]. Further, CD45RA expression was found to distinguish multipotent (CD133+ CD34+ CD45RA−) from lympho‐myeloid (CD133+ CD34+ CD45RA+) human HPC fractions [44]. Yet, a CD133+ CD34+ population could be detected also in endothelial colony-forming progenitor cell preparations from steady-state peripheral blood leukapheresis indicating that this phenotype may not be exclusively assigned to HSCs [45]. Moreover, different levels of CD34 expression separate human HSC and HPC subsets with regard to their metabolic activity and stemness [14, 46, 47].

Interestingly, this considerable heterogeneity of hematopoietic stem and progenitor cell populations is not only restricted to the postnatal organism, but can also be found in the fetus where the glycosylphosphatidyl inositol-anchored protein (GPI)-80 identifies a subset of human fetal liver HSPCs with self-renewal capacity (no megakaryocytic cells reported) [48], and in human cord blood GPI-80 is a marker of immature CD34+/− SRCs [49]. Thus, the above mentioned hierarchical hematopoiesis concept may not completely illustrate the degree of HSPC heterogeneity, particularly considering more recent findings that highlight their molecular and functional diversity [23]. These studies suggest an earlier lineage commitment [50] (“early split model”), or do even propose to replace the hierarchical model with various (progenitor) cell states defined by their transient transcriptome signatures (“continuous Waddington-like model”) [23]. Particularly, the latter gives room to address the variable transplantation outcomes for individual HSC clones and to implement the concept of HSC lineage biases, i.e. favored (due to lineage priming) differentiation into distinct cell types, but retaining multi-lineage potential [23]. This is supported by a recent study reporting a murine HSC subset that highly expressed the junctional adhesion molecule 2 (Jam2). These Jam2high HSCs featured a greater T cell reconstitution capacity, and their frequency was impacted by hematopoietic stress such as T cell depletion or fluorouracil or byphenylhydrazine injection [51].

Novel technologies such as single-cell transcriptomics combined with machine learning are expected to broaden our understanding of hematopoiesis including HSPC biology in situ and ex vivo. Recently, single-cell RNA sequencing of human HSPCs derived from pluripotent stem cells revealed, together with an artificial neural network that was trained on human fetal liver cell phenotypes, CD44, CD326, ICAM2/CD9, and CD18 as markers of immature and lineage-committed HSCs and progenitor cells including a novel HSC subset that was not identified before in studies analyzing selected cell populations [52].

Currently, the following drivers of HSPC heterogeneity are discussed: mutations causing functional effects, inconsistent impact on epigenetic regulators, changing metabolic activity, uneven distribution of cell components during cell division, oscillations of transcriptional activity, and not least, the microanatomical localization within the niche where HSCs and progenitors interact with various niche cell types and are exposed to biophysical conditions that could impact their biology (e.g. oxygen partial pressure, pH) [23]. Stressors such as BM damage, bleeding, or treatment with thrombopoietin or granulocyte colony-stimulating factor (G-CSF) (see below) can, via cytokine signaling, shift the dormant HSC to the active HSC phenotype as shown in mouse studies (no megakaryocytic cells reported [12, 34, 53] except [54]). This can be reversed when homeostasis is re-established [12] (Fig. 1). A recent study showed that stress-induced repeated HSC activation can lead to DNA damage in HSCs eventually obliterating hematopoiesis in mice [54]. Regarding mutations the concept of clonal hematopoiesis has been developed which is associated to malignant and nonmalignant diseases [55, 56]. Such clonally expanding stem/progenitor cells can affect the original stem cell pool, or even take over hematopoiesis completely in humans [57] eventually decreasing its heterogeneity.

**Fig. 2:**
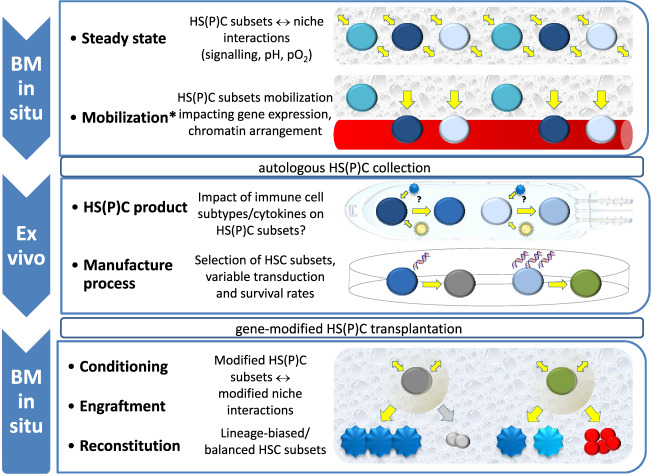
Drivers of HSPC heterogeneity at distinct stages of HSC-based gene therapies. HSPC heterogeneity is relevant, likely to variable degrees, in all phases of HSC-based gene therapies: in the BM niche in vivo before HSPC collection: disease-specific effects on HSPC subsets that interact with the nice; during HSPC mobilization (*does only pertain to PBSC collection, but not to BM collection): preferred mobilization of HS(P)C subsets; ex vivo during HSC selection/enrichment and manufacture of the gene therapeutic: interactions with immune cells and cytokines possibly impact HS(P)C subsets and manufacture protocols select for HSC subsets with variable transduction and survival rates; in situ again: interactions of modified HS(P)C subsets with the niche that has been altered by the conditioning regime could affect engraftment and long-term reconstitution.

As outlined above, both PBSCs and BM are sources for HSC-based gene therapies. PBSC products contain more total CD34+ cells compared to BM, as well as more MPP and erythro-myeloid precursors [58]. Moreover, PBSC products contain more T cells [59], but interestingly with a lower proportion of regulatory T cells [60]. Moreover, HSPCs in the human blood, and thus also likely in the PBSC product, are exposed to higher levels of proinflammatory cytokines compared to HSPCs in the BM [61]. It is reasonable to assume that the immune cell subtypes and cytokines in these products could impact HSC subsets with to date unclear consequences for HSC-based gene therapy manufacture. Additionally, progenitor cells that might be needed to bridge the time until robust HSCs engraftment, could be affected as well. Moreover, such interactions including susceptibility to gene transfer might vary individually, thus further challenging the standardization of the production process (Fig. 2). To address these issues it might be useful to assess the genetic modifications for each cell type being present in the graft, and/or to develop manufacture concepts that target a specific cell type. In fact, high efficiency human LT-HSCs gene editing using CRISPR/Cas9 technology was recently reported [43].

Another study showed that the immature human CD34+ 133+ 90+ HSC subset had the lowest transduction rates and this subset also disappeared during ex vivo culturing, but optimization of the manufacture protocol (modulating gene delivery technology and cell culture conditions) improved transduction efficiency and long-term engraftment (no megakaryocytic cells reported) [62]. It appears obvious that, on single-cell level, each vector-transduced HSC carries an individual integration site signature. Referring to the above mentioned heterogeneity of HSC subsets, this, on the one hand, imposes a tremendous challenge for standardized gene therapies production and reliable projections of desired and undesired effects. On the other hand, considering that the individual signatures are passed on to the hematopoietic lineage cells derived from the each individual HSC, this technology was successfully applied to study the fate of transduced human HSCs and their progeny after transplantation in vivo specifically identifying distinct HSC differentiation schemes [63].

### The roles of the niche

In the BM the HSCs reside mainly (80%) in the endosteal niche [64], and combined single-cell and spatially resolved transcriptomics delivered a high resolution picture of the BM cell composition including HSPCs and reticular cell subsets highly expressing CXCL12 where the latter were found in microanatomically different niche regions (endosteal, sinusoidal, and arteriolar) and support perivascular „micro-niches“ in mice [65]. The CXCR4-CXCL12 axis is a highly relevant communication link between HSCs and the niche as shown in a mouse model [66]. Thus, it is reasonable to assume that micro-niche specific signaling between niche cells and HSC subsets, including genetically modified HSPCs, is highly relevant for their mobilization, engraftment, and long-term function. The transplanted HSCs and progenitors, challenged by oxidative stress, need to home to the reconstituting niche in the BM being damaged by the conditioning treatment (myeloablation) [67], or other stressors [25]. Further, complex interactions with niche cells result in robust engraftment of the transplanted HSCs which exit from quiescence and start the production of new blood cell progeny [67] that carry the HSCs’ genetic information.

As outlined above, niche signaling does not only contribute to HSC maintenance and heterogeneity, but may also affect HSPC lineage priming (no erythroid, or megakaryocytic cells reported) [23, 68]. Specifically, cytokines and cytokine receptor expression on HSCs and progenitor subsets as well as their interactions with niche cells impact the direction of lineage differentiation [23]. Lineage commitment was shown to be developmental, and is also controlled by the niche where BMP and TGFb signaling pathways play opposite roles [39, 68, 69].

Moreover, the relevance of the conditions in the BM niche where the genetically modified HSCs are expected to engraft is evident as before transplanting the patient undergoes myeloablative treatment [5]. The impact of the BM conditioning regime on the niche is highlighted by a recent mouse study showing that after irradiation or anti-c-kit antibody treatment only few HSC clones contributed to hematopoietic reconstitution after transplantation featuring unbalanced differentiation (no erythroid, or megakaryocytic cells reported) [70]. In contrast to the BM niche the likelihood to detect HSPCs in the peripheral blood under steady-state conditions is very low. This is illustrated by the low frequency of cells expressing CD34 in the human blood (three cells per µL blood [71]). As outlined above this marker does not sufficiently identify HSCs. Thus, to increase the chance to collect sufficient numbers of HSCs from the blood, they need to be mobilized from the BM niche into the peripheral blood. We will next discuss HSC mobilization as another variable that impact HSC-based gene therapies.

### HSPC mobilization effects

To date several substances are approved for HSPC mobilization: granulocyte-macrophage colony-stimulating factor (GM-CSF), G-CSF, and plerixafor (AMD3100) [16]. G-CSF and GM-CSF, acting via their respective receptors on monocytes/macrophages and neural cells in the niche, do not only stimulate granulopoiesis, but induce also signaling that leads to loss of osteoblasts and reduced expression of CXCL12, VCAM-1, and stem cell factor on mesenchymal stromal cells (MSCs) which eventually results in the release of HSPCs from the niche into the peripheral blood [72]. Plerixafor specifically blocks the interaction of CXCR4 on HSPCs with its ligand CXCL12 on MSC and osteoblasts hereby mobilizing the HSCs into the blood. Currently, G-CSF is most commonly used for HSPC mobilization (GM-CSF is no longer available in some countries [73]). Besides other CXCR4 antagonists than plerixafor, further mobilization agent developments are underway targeting HSC-niche cell interactions such as inhibiting integrin α4β1 on HSCs [72], or stimulating chemokine receptor CXCR2 on neutrophils (mouse study, no erythroid cells reported) [74]. These mobilizing agents can enhance the HSPC frequency in the peripheral blood about 40 times allowing collections of more than 10 million CD34+ cells per kg of the recipient’s body weight [71], where plerixafor acts faster and is more effective when combined with G-CSF [72]. But are there individual effects of mobilization agents on HSC? Indeed, various studies in humans, nonhuman primates, and rodents reported different mobilization effects on distinct HSC populations. The combinatory application of plerixafor with G-CSF can not only increase the total yield of CD34+, but specifically immature CD34 + CD38low phenotpyes, and plerixafor is specifically potent to mobilize long-term culture-initiating cells (LTC-ICs) and SRCs compared to G-CSF (Table 1). Additionally, HSPC mobilized with plerixafor feature different miRNA and cDNA expression signatures compared to HSPCs mobilized with G-CSF [72].

**Table 1:** Mobilization effects on HSPC populations.

	Plerixafor	G-CSF
Peak of CD34 + HSPC mobilization [72]	6–10 h	Day 4–5
CD34 + HSPC mobilization potential [72, 124]	G-CSF combined with plerixafor > single use G-CSF > single use plerixafor	
Mobilization of HSPC phenotypes [72, 124]	CD34 + CD38lowCD133 + HSCs: G-CSF combined with plerixafor > single use G-CSF	
	CD34 + CD38lowCD90 + HSCs: G-CSF combined with plerixafor > single use G-CSF	
	CD34dimCD45RA + CD123hiCXCR4hi plasmocytoid DC precursor cells ↑	CD34 + CD45RA − CD123 + / − HSPC↑
Content of BFU-E and CFU-GEMM in the graft [72]	↓	↑
Content of long-term culture-initiating cells (LTC-ICs) in the graft [72]	↑	↓
Content of SCID-repopulating cells (SRCs) in the graft [72, 125]	↑	↓
Proportion of CD34 + HSPCs in the G1 phase of cell cycle [72]	↑	↓
Percentage of CXCR4 + CD34 + HSPCs in the graft [72]	↑	↓
Percentage of integrin α4β1 + CD34 + HSPCs in the graft [72]	↑	↓
Content of DCs in the graft [72]	G-CSF combined with plerixafor > single use G-CSF	
Content of T cells, B cells, and NK cells in the graft [72]	↑	↓

Is there evidence on the impact of HSC mobilization on HSC-based gene therapy? Wiskott–Aldrich syndrome protein (WASp)-deficient HSCs could be sufficiently mobilized with G-CSF and transduced effectively with a lentiviral vector without impairing their engraftment, yet, WASp deficit in HSCs, and probably also in the niche, dramatically decreased B cell reconstitution in patients [75]. In another study, CD34+ cells mobilized from patients with β-thalassemia with plerixafor plus G-CSF featured fewer lentivirus vector-positive colonies, i.e. lower gene transfer efficiency, than CD34+ cells that were mobilized with plerixafor alone [76]. Yet, despite their lower transduction efficiency, these CD34+ cells being mobilized with plerixafor combined with G-CSF produced higher β-globin expressing erythroid cells per lentivirus copy number, and showed superior early engraftment in patients compared to cells that were mobilized with either G-CSF or plerixafor alone [76]. Mechanistically, this may go in line with the above mentioned impact of mobilization agents on HSPC gene expression and/or chromatin arrangement, and it is reasonable to assume that hereby variable vector integration sites occurred in the genome with different functional effects [76]. These findings illustrate that the mobilization regime is another relevant variable depending on the disease, and warrants consideration for the development of HSC-based gene therapy concepts.

### How HSC heterogeneity impacts gene therapy of specific diseases

As discussed above studies in mice and nonhuman primates are pointing toward the existence of lineage (myeloid-, lymphoid-, megakaryocytic)-biased [77–79] and balanced HSCs which are even maintained following HSCT [50, 80]. A comprehensive identity profiling of all human HSC subsets has not been delivered yet. However, a study reported an age-related increase of myeloid-biased HSCs in the human BM (no T-cells, erythroid, or megakaryocytic cells reported) [27]. Another study described, applying a barcode-based clonal tracking analysis of several gene therapy patients, distinguishable balanced, myeloid-biased and T-cell biased HSCs with their own unique pattern of integration sides in humans [63]. Even though this effect might be caused by the gene therapy treatment per se, the existence of biased HSCs was suggested in human cord blood where myelo-lymphoid lineage restriction may occur already in the HSC compartment before the emergence of lymphoid-primed MPPs [47]. Yet, the biased HSCs discussed above could turn out as long-lived progenitors being lineage-committed to megakaryocytes, megakaryocyte-erythroid cells, or common myeloid cells, as shown in a previous mouse study [78].

Nevertheless, HSC gene modification concepts must consider that different blood disorders affect specific hematopoietic cell types [18, 39]. We will discuss the relevance of HSCs heterogeneity for gene therapies in exemplary diseases with a specific emphasis on the potential key target HSC types, i.e. myeloid-biased, lymphoid-biased, and balanced HSCs.

### β-Thalassemia and sickle cell diseases (myeloid-biased HSCs)

Hemoglobin disorders account for about 3.4% of deaths in children younger than 5 years. Approximately 80% of the children with an hemoglobin disorder are affected by sickle cell disease (SCD) whereas 20% suffer from various forms of thalassemia. Globally speaking SCD is the most common monogenetic disorder [81]. Each year ~300,000 children are born with SCD [82], that is defined as homozygosity for the *sickle hemoglobin* (*HbS*) gene, a missense mutation [Glu6Val, rs334] in the *β-globin* gene leading to a deoxygenation-induced HbS polymerization. This primary event causes a multisystem disorder driven by RBC sickling, causing an increase in blood viscosity, vaso-occlusive crises, and hemolysis [83]. Moreover, 68,000 children are born with various thalassemia syndromes each year [84]. As another β-hemoglobinopathy, β-thalassemia is characterized by reduced or absent β-globin chain synthesis, originating form a heterogeneous set of autosomal recessive inheritances. The inability to build β-chains leads to ineffective erythropoiesis, intramedullary hemolysis, and hemolytic anemia [85]. Both above mentioned hemoglobin disorders are usually treated with blood transfusions and hydroxyurea, and allogeneic HSCT was the only available curative therapeutic option. In February 2013 Betibeglogene autotemcel (LentiGlobin BB305), a lentiviral vector which ex vivo inserts a functioning version of the *HBB* gene (BA-T87Q-globin) into a patient’s HSPC was granted an orphan drug status by the European Medicines Agency (EMA) and by the U.S. Food and Drug Administration (FDA). In 2015 it was given breakthrough therapy designation by the FDA and approved for medical uses in the European Union in May 2019. To date, this is the only curative therapeutic option for adults and adolescents 12 years and older with transfusion depended β-thalassemia who do not find an HLA-matched HSC donor, but are eligible for autologous HSCT. Another patient suffering from SCD was treated with the same LentiGlobin BB305 vector resulting in the complete correction of the clinical phenotype. Notably, this patient developed therapeutic β-globin levels around 50%, became transfusion independent, and had a stable clinical profile similar to those of heterozygous *HbS* carriers [6]. In another trial (HBG206 study) focusing on SCD in adults a lower in vivo vector copy number was detected and a lower cell dose of transduced CD34+ cells per kg body weight could be re-infused. Here, only a low transduction level could be achieved, and no clinical benefit in patients with SCD was found, highlighting the importance of transduction efficiency of the genetically modified autologous grafts and the procurement method to increase cell doses [86]. As outlined above, in gene therapy with human HSC, it is feasible to monitor each gene-corrected cell and its progeny by the sites of their integrating vectors, barcoding them in a unique way [63]. This enabled in vivo lineage tracing by sampling blood cells and using DNA sequencing to identify the vector integration sites. In the first gene therapy trial for β-thalassemia (LG001 study) a patient with severe, transfusion-dependent βE/β0-thalassemia was monitored for 3 years after reinfusion of autologous CD34+ cells transduced with a lentiviral vector expressing a common, tagged β-globin, resulting in complete transfusion independence. Deep-sequencing analysis revealed the dominance over time of an integration site (IS) at the high-mobility group AT-hook 2 (HMGA2) in both granulocytes (qPCR analysis revealed that approximately half of the vector-bearing WBC were carrying the HMGA2 IS) and erythroblasts, but was not found in lymphocytes. Nevertheless, overall non-transduced cells continued to predominate, so that only a small portion of circulating cells and clonogenic progenitors were HMGA2 IS positive (<10%). In the years after transplantation the IS HMGA2 clone continuously contributed to hematopoiesis without any detectable pathological aberrations. The stable detectability of the HMGA2 IS in similar numbers of erythroblasts, granulocytes, but not in lymphocytes, suggests the conclusion that a fraction of the hematopoietic progeny after lentiviral transduced autologous HSCT originated from a myeloid-biased long-term HSC in this patient [87]. Instead of hoping for a randomly occurring stochastic transduction event the targeted identification of distinct myeloid-biased subsets in autologous HSC and the selectively expansion of these subsets could lead to a more precise, disease-tailored and an increased transduction efficacy in viral vector-driven gene therapies as mentioned above. This might be true as well for a currently investigated therapy strategies using the CRISPR/Cas9 system. BCL11A as a developmental stage-specific repressor plays a significant role in the repression of fetal hemoglobin [88, 89]. Regaining the synthesis of fetal hemoglobin in significant amounts can improve or even heal the clinical state of patients with β-hemoglobinopathies [90]. Recently, one patient with SCD and one patient with β-thalassemia were treated successfully with the novel drug CTX001, an ex vivo CRISPR/Cas9 system editing the BCL11A region of patient’s HSPCs [91]. Remarkably, both patients became transfusion independent, and 18 months after CTX001 infusion, the patient suffering from transfusion-dependent β-thalassemia had total hemoglobin levels of 14.1 g/dL, 13.1 g/dL fetal hemoglobin, and 100% RBCs expressing fetal hemoglobin [91]. Nevertheless, off-target effects are a valid concern regarding gene editing techniques, such as the CRISPR/Cas9 system [92], and the future will show the safety profile of this approach. Specifically, variations in the human genome were shown to affect on- and off-target sites in CRISPR-based therapies [93]. Furthermore, there is strong evidence that broad knockdown of BCL11A in all human and mouse CD34 + HSPCs might lead to engraftment failures [94–96], whereas a lineage-specific knockdown might avoid this phenomenon [97], and more precise approaches targeting specific HSC subsets also regarding the number of gene-edited cells might improve safety. As a concept that may strike a new path towards lineage-specific gene therapy in HSCs a current clinical trial (NCT03282656) is investigating a lineage-specific approach using a lentiviral vector that mediates erythroid-specific knockdown of BCL11A via RNA interference using a microRNA-adapted short hairpin RNA [98].

### Wiskott–Aldrich-syndrome (balanced HSCs)

Contrary to the β-hemoglobinopathies, in which only a subset of the myeloid-lineage is affected, the defect of the WASp effects all hematopoietic lineages. The WAS is an X-linked recessive disease characterized by the classic triad of recurrent infections, thrombocytopenia, eczema, and autoimmunity [99, 100]. WASp is a key regulator of actin polymerization in hematopoietic cells, required for cytoskeletal (re)organization, signal transduction, cell locomotion, terminal differentiation, and immunologic-synapse formation [101]. Regarding the great relevance of the WASp in hematopoietic cell biology a defect in this protein consequently results in multiple dysfunctions of T and B cells, NK-cells, and impaired migratory responses in all leukocyte subgroups, as well as decreased production of platelets and an increase of their destruction [102, 103]. Previously, only HSCT had offered hope of cure. The first attempts for autologous gene therapy in WAS using a gammaretroviral vector led to a stable integration of the corrected gene, and the expression of WASp decreased bleeding, infection, and autoimmunity [104]. Nevertheless, insertional oncogenesis occurred which lead to the development of leukemia and myelodysplastic syndrome in 8 out of 9 patients after several years [105]. The use of lentiviral vectors led to more promising results. In the three treated patients stable and durable integration of the corrected WASp gene and the expression of WASp was noted, and the patients showed significant clinical improvements without insertional oncogenesis-associated events [4, 106]. In a recent study including four WAS, one SCD and one β-thalassemia patient after autologous HSC-based gene therapy, clonal tracking by IS analysis, as described as above, added strong evidence for the existence of lineage-biased HSCs [63]. In addition to the identification of myeloid-, lymphoid-biased and balanced HSCs, the group also suggested the existence of a T-cell-biased HSC subpopulation. Another study also tracking IS in patients with WAS postulated the long term persistence of lymphoid progenitor cells after gene therapy [107]. Defined lymphoid- or even T-cell-biased lineages derived from gene-modified HSCs may open up new possibilities for the treatment of other immunodeficiencies.

### X-linked severe combined immunodeficiency (X-SCID) (lymphoid-biased; T-cell-biased)

30–40% of all SCIDs are classified as X-SCID which is caused by a mutation in the common gamma chain (γc) -encoding gene [108]. The γc chain is part of many cytokine receptors like IL-2, IL-4, IL-7, Il-15 and Il-21, and a γc chain defect blocks T- and natural killer cell differentiation [109]. Missing the Th2-cell stimulus B lymphocytes do not produce sufficient immunoglobulin levels. Therefore, patients witch SCID do often develop multiple viral and fungi infections early in life. As for many monogenetic hematopoietic disorders allogeneic HSCT was the only curative treatment also for X-SCID. To monitor the in vivo ability of the thymus for thymopoiesis T-cell receptor (TCR) excision circle (TREC) assays can be used, to detect episomal DNA circles generated during TCR genes rearrangement [110]. The sustained presence of circulating T-cells in patients 10 years after gene-corrected HSCT with normal TREC levels was documented. Despite the fact that 6–10 years after gene therapy transduced T-cells were still circulating, transduced B-lymphocytes and myeloid cells were not detected [3]. This suggests that either the B cells and myeloid cells or their precursors did not survive, or that only T-cell-biased HSCs were successfully transduced and showed long-term engraftment.

### Bare lymphocyte syndrome type 2 (lymphoid-biased; T-cell-biased)

The bare lymphocyte syndrome type 2 (BLS II) is a rare recessive genetic condition in which the major histocompatibility complex II (MHC II) genes are not expressed, leading to an immune system that cannot fight infections effectively [111]. To date, mutations in one of the four genes have been identified as cause of the disease: class II trans-activator, regulatory factor of the Xbox5, RFX-associated protein (RFXAP), and RFX ankyrin repeats [111, 112]. As these genes code for transcription factors, the pathogenic mutations are not located in the gene for the MHC II itself, but only in the transcriptional factors regulating the expression of the MHC II gene [111, 112]. Clinically BLS II comes as SCID, but does not lead to decreased B- and T-cell counts, because the development of these cell types is not affected. So far, the only available treatment is HSCT, but gene therapy might be an interesting option [113]. It was demonstrated for peripheral blood-derived lymphocytes from a patient with BLS II (but not for HSCs) that an RFXAP-lentiviral vector restored the expression of MHC II in 48% of these cells in vitro [113]. Focusing specifically on the T-cell-biased HSC population regarding gene therapy in BLS II could have a substantial beneficiary impact.

### Acquired immune deficiency syndrome (lymphoid-biased; T-cell-biased)

Another disease in which T-cells are in focus is the acquired immune deficiency syndrome (AIDS) caused by the human immunodeficiency virus (HIV) 1 and 2. The invasion and replication of the virus inside CD4 + T helper cells decreases not only their numbers dramatically, but eventually leads to the destruction of the immune system. Even though therapeutic options increased during the last 30 years a cure has still not been found. The CCR5 protein is a relevant co-receptor of CD4 that HIVs need to enter the T helper cells [114]. It is known that individuals born with a homozygote defect in the *CCR5* gene, usually a deletion of 32 amino acids (Delta32), are mostly immune to infections with the HIV [115–117]. It was reported in 2009 that first cured HIV patient (“Berlin patient”) was treated in 2007 by an allogenic CCR5 Delta32/Delta32 HSCT [118]. In 2019 another HIV patient (“London patient”) went successfully through the same procedure. Thirty months after analytical treatment interruption the HIV-1 viral load in the plasma of the “London patient” remained undetectable [119]. The first successful gene editing strategy in humans regarding HIV was published in 2014 [120]. Using zinc finger nucleases CCR5 receptor modified CD4 + -T-Cells were infused into HIV patients. After complete treatment, the viral load in the patients decreased and in one patient HIV even disappeared. Using autologous lymphoid-biased HSCs or T-cell biased HSCs could further improve clinical outcome regarding gene editing strategies in AIDS. The same might be true for a recently published approach: Xu et al. [121]. transplanted CRISPR-edited CCR5-ablated allogeneic HSPCs to a patient with HIV and acute lymphocytic leukemia (ALL). Due to a low editing efficiency, the proportion of CCR5 ablation ranged between 5.2 and 8.3% during the 19-month long-term engraftment. The percentage of CD4+ cells with CCR5 ablation increased by a small degree during antiretroviral-therapy interruption. Nevertheless, the percentage of CCR5 disruption in lymphocytes was only ~5% even though the ALL was in full remission and with full donor chimerism. Notably, no lineage specificity of the treatment was observed. The editing efficiency in other hematopoietic lineages was similar to these of the lymphocytes, or slightly higher. Those results showed that CRISPR-edited HSCs successfully engrafted and differentiated into multiple lineages that retained the gene editing, and focusing on lineage-biased HSCs might improve the outcome and clinical benefit.

## Outlook and conclusion

To date, there are only limited data about the long-term success of HSC-based gene therapies. Not only with regard to treatments in early childhood, sustainable life-long effects are desired. So far, it is not clear whether this goal could be achieved with the current treatment concepts. Only a minute fraction of transduced CD34+ cells do engraft and can sustainably maintain hematopoiesis (LT-HSCs) [4]. Future studies may address the question if the therapeutic effect of HSC-based gene therapies could be improved and/or prolonged by a combined approach targeting, i.e. specifically deliver sufficient vector copy numbers, LT-HSCs as well as lineage-biased HSC subsets that are relevant for the respective disease. Here, successful strategies would need to include uniquely defining surface protein signatures that precisely define the respective HSC subsets to be targeted. As outlined above, LT-HSCs come at low frequency within the CD34+ fraction. Thus, fewer cells might have to be genetically engineered hereby likely reducing the manufacturing costs. Yet, exclusively focusing on HSCs (subsets) as gene therapy targets could bear the risk to neglect potentially critical progenitors that may be needed to reduce the time until sustainable HSC engraftment [17]. This could be mitigated by co-transplanting unmodified progenitors [122]. Depending on the pathophysiology of the respective disease, e.g. SCD vs. WAS, the co-transplanted progenitors might need genetic modification as well.

There is already evidence that targeting HSC subpopulations for gene therapy could be a promising strategy for the future as demonstrated by CD133-targeted gene transfer into HSCs from a patient with the X-linked form of chronic granulomatous disease that resulted in a higher long-term HSC repopulating rate compared to untargeted gene transfer [38]. Utilizing the CD150 surface marker, or the Hoechst dye efflux, made it possible to prospectively enrich both myeloid- and lymphoid-biased murine HSCs (no erythroid and megakaryocytic cells reported) [123]. Furthermore, myeloid-biased HSCs and lymphoid-biased HSCs can be distinguished by their different response to cytokines such as transforming growth factor β and interleukin 7 [69, 77]. Exploring the effects of such cytokines in detail and their applicability might help to develop lineage-specific HSC enrichment protocols. Additionally, off-target effects of current gene editing technologies pose are a relevant issue to be resolved to minimize the risk for side effects. Last but not least, the observed impact of different conditioning regimes on lineage biases after transplantation in mice [70] warrants further investigation, and optimizing these could add value to the therapeutic potential of gene therapies.

Altogether, improved understanding of HSC heterogeneity might help to increase transduction efficiency in viral vector gene therapies, increase gene editing efficiency in disease relevant cells, reduce the risk of off-target effects for gene editing systems, and reduce side effects in hematopoietic lineages that are irrelevant for the disease.
